# The performance of a Bayesian value-based sequential clinical trial design in the presence of an equivocal cost-effectiveness signal: evidence from the HERO trial

**DOI:** 10.1186/s12874-024-02248-9

**Published:** 2024-07-19

**Authors:** Charlie Welch, Martin Forster, Sarah Ronaldson, Ada Keding, Belen Corbacho-Martín, Puvan Tharmanathan

**Affiliations:** 1 York Trials Unit, Department of Health Sciences, University of York, Heslington, York, YO10 5DD UK; 2 Department of Statistical Sciences ‘Paolo Fortunati’, University of Bologna, Bologna, Italy

## Abstract

**Background:**

There is increasing interest in the capacity of adaptive designs to improve the efficiency of clinical trials. However, relatively little work has investigated how economic considerations – including the costs of the trial – might inform the design and conduct of adaptive clinical trials.

**Methods:**

We apply a recently published Bayesian model of a value-based sequential clinical trial to data from the ‘Hydroxychloroquine Effectiveness in Reducing symptoms of hand Osteoarthritis’ (HERO) trial. Using parameters estimated from the trial data, including the cost of running the trial, and using multiple imputation to estimate the accumulating cost-effectiveness signal in the presence of missing data, we assess when the trial would have stopped had the value-based model been used. We used re-sampling methods to compare the design’s operating characteristics with those of a conventional fixed length design.

**Results:**

In contrast to the findings of the only other published retrospective application of this model, the equivocal nature of the cost-effectiveness signal from the HERO trial means that the design would have stopped the trial close to, or at, its maximum planned sample size, with limited additional value delivered via savings in research expenditure.

**Conclusion:**

Evidence from the two retrospective applications of this design suggests that, when the cost-effectiveness signal in a clinical trial is unambiguous, the Bayesian value-adaptive design can stop the trial before it reaches its maximum sample size, potentially saving research costs when compared with the alternative fixed sample size design. However, when the cost-effectiveness signal is equivocal, the design is expected to run to, or close to, the maximum sample size and deliver limited savings in research costs.

**Supplementary Information:**

The online version contains supplementary material available at 10.1186/s12874-024-02248-9.

## Introduction

There is increasing interest in the use of adaptive designs to improve the efficiency of clinical trials. Such designs monitor outcome data as they arrive over the course of the trial, so that planned design changes can be made in response to accumulating evidence [1–8]. There is also growing interest in using clinical trials to examine the cost-effectiveness of the technologies under investigation, alongside their clinical effectiveness, with the objective of assessing their ‘value for money’ to the health care system [9, 10]. However, relatively little work has investigated how economic considerations – including the cost of carrying out a clinical trial – might inform the design and conduct of adaptive clinical trials.

Recent NIHR-funded research initiatives in the United Kingdom – notably the ‘EcoNomics of Adaptive Clinical Trials’ (ENACT) and the ‘Costing of Adaptive Trials’ (CAT) projects^1^ – have sought to address this gap in the literature. In this paper, we focus on one of the principal outputs of the ENACT project; a retrospective application of a recently developed Bayesian value-based sequential clinical trial design [13, 14] to data from the ‘Hydroxychloroquine Effectiveness in Reducing symptoms of hand Osteoarthritis’ (HERO) trial [15–17].

The HERO trial was a fixed sample size, non-sequential clinical trial designed according to frequentist principles. It recruited and randomised a fixed, predetermined number of patients to its two arms, collected data on a key primary clinical endpoint and tested a null hypothesis positing that the experimental treatment (hydroxychloroquine) was no better than placebo for the treatment of hand osteoarthritis (OA) with respect to this endpoint. The sample size was chosen to target 80% power for this hypothesis test. In this paper we investigate: (1) what would have happened had the HERO trial been conducted as a Bayesian value-based, sequential, clinical trial; (2) how much additional value such a design might have delivered to the health care system, over and above that delivered by a non-adaptive design and (3) how multiple imputation methods for missing data can be incorporated into the implementation of the value-based sequential model.

The sequential model that we investigate permits the clinical trial to stop short of its maximum sample size through explicit consideration of the trade-off between the benefits and costs of continuing the trial. As we discuss below, the sequential trial’s maximum sample size can be chosen to be equal to, smaller than, or greater than the sample size that is required for a traditional, frequentist, fixed sample size design. In contrast to notions of efficiency considered by most proposed sequential designs, where the objective is to reduce the expected sample size of a trial (subject to some constraints), the objective of the value-based sequential model is to maximise the overall expected net benefit of the trial and subsequent treatment adoption recommendation to the health care system. As our results show, a value-based approach could motivate a sample size that exceeds that which would be planned for a traditional, frequentist, fixed sample size clinical trial.

To date, the published literature contains only one other retrospective application of this model: [18] applied it to the ‘PROximal Fracture of the Humerus: Evaluation by Randomisation’ (ProFHER) pragmatic trial [19] and found that the design could have reduced the number of patients randomised by an estimated 14% (saving about 5% of the research budget), while at the same time resulting in an adoption recommendation which was consistent with that of the actual trial. A bootstrap analysis investigating the performance of the model ‘on average’ suggested a reduction in expected sample size of approximately 38% (compared with a fixed length design), an estimated 13% saving in the research budget, and an estimated probability of 0.92 of an adoption recommendation consistent with that of the actual trial.

These results were driven by a relatively strong cost-effectiveness signal in favour of one of the two interventions that were under investigation. In contrast, the HERO trial’s cost-effectiveness evidence was much less clear-cut, with neither of the treatments showing a clear cost-effectiveness advantage over the other. The data from the HERO study therefore provide an ideal opportunity to assess the value-based sequential model’s performance in the presence of an equivocal cost-effectiveness signal. In doing so, we note that our focus in this paper is not on whether the Bayesian sequential rule that is proposed could replace a frequentist fixed sample size or group sequential design. Instead, our interest is whether the model could complement existing designs, by providing additional information to trials teams about whether or not interim evidence suggests that the expected benefit of continuing the trial outweighs the expected benefit of stopping it.

The rest of this paper is structured as follows. In the Methods section we provide an overview of the value-based sequential model and the HERO trial, and describe in detail the application of the former to the latter. In the Results section we report the quantitative findings of our application. The Discussion section discusses our results, compares them with those from the ProFHER application and considers directions for future research.

## Methods

### The Bayesian value-based model of a sequential clinical trial

In this section we provide an intuitive account of the Bayesian value-based sequential model that we apply to the HERO trial. Full details may be found in the two papers which state and solve the model [13, 14].

Consider a randomised clinical trial in which a new health technology, *N*, is to be compared with a control, or standard, technology, *S*, on cost-effectiveness grounds. Patients are randomised sequentially, and in a pairwise manner, to the two arms of the trial and outcome and treatment cost data are measured over a follow-up period of defined length. The outcome of interest is whether technology *N* is a cost-effective choice for the reimbursement agency responsible for funding the health technology, where cost-effectiveness is measured in terms of incremental net monetary benefit (INMB). Label the pairwise allocations as $i = {1},\ldots ,T_{\max }$, where $T_{\max }$ is the maximum number of pairwise allocations that can be made. Define the net benefit of technology *j* for pairwise allocation *i* as $\text {NB}_{ij} = \lambda E_{ij} - C_{ij}$, $j \in \{N,S\}$, where the random variables *E* and *C* denote effectiveness and cost, respectively, and $\lambda$ denotes the reimbursement agency’s maximum willingness to pay for one unit of effectiveness (as an example, in the HERO trial, *E* is a Quality Adjusted Life Year (QALY), so $\lambda$ could be the UK National Health Service’s valuation of one QALY, generally taken to equal between £20,000 and £30,000 [20]).

Define the incremental net monetary benefit of the new technology versus the standard for pairwise allocation *i*, denoted hereafter as $X_i$, as the net benefit of *N* minus the net benefit of *S* for allocation *i*:

1 $$\begin{aligned} X_i = \text {NB}_{iN} - \text {NB}_{iS} = \lambda (E_{iN} - E_{iS}) - ( C_{iN} - C_{iS} ). \end{aligned}$$

The $X_i$ are assumed to have a normal distribution with unknown expected value $\mu _X \equiv \mathbb {E}[X]$, but known variance $\sigma ^2_X$ (the assumption of normality of the data is something that could be tested during the course of the trial, and is something we carry out in our application). Taking a Bayesian perspective, prior beliefs about $\mu _X$ are modelled using a normal prior distribution with expected value and variance equal to $\mu _0$ and $\sigma ^2_0$, respectively. These values can be informed by existing evidence concerning the two technologies, a pilot study, or expert opinion, with limited or unreliable prior evidence being represented by a ‘diffuse’ prior distribution with expected value close to, or equal to, zero.

As the trial progresses, measurements of incremental net monetary benefit arrive sequentially from pairs of patients who have been followed-up and Bayes’ rule is used to obtain successive posterior distributions for $\mu _X$. Under the assumptions of the model, namely that the prior distribution is normal and the data and associated likelihood function are normal, the posterior distribution is also normal. After *n* pairwise allocations have been observed, the posterior mean and variance for $\mu _X$, denoted $\mu _n$ and $\sigma ^2_n$ respectively, are given by standard expressions [21]:

2 $$\begin{aligned} \mu _n = \frac{n_0 \mu _0 + n \bar{x}}{n_0 + n},\,\,\,\sigma ^2_n = \frac{\sigma ^2_X}{n_0 + n}, \end{aligned}$$

where $n_0 = \sigma ^2_X/\sigma ^2_0$ is the prior’s so-called ‘effective sample size’ and $\bar{x}$ is the sample average of the *n* observations of INMB.

The objective of the model is to define a policy, or rule, that determines whether, conditional upon the observed data and hence the resulting posterior distribution, recruitment to the trial should stop, or another pair of patients should be recruited and randomised. The policy maximises the expected net benefit of the trial and subsequent technology adoption decision, defined as the difference between the expected benefit accruing to the *P* patients whose treatment will be determined by the adoption of the superior technology once the trial concludes, minus any costs incurred in switching technologies, minus the expected cost of carrying out the trial. The policy takes the form of a stopping boundary in ($n\,\,\times$ prior/posterior mean space) which indicates that recruitment should continue if the posterior mean for $\mu _X$ lies within the area enclosed by the stopping boundary and recruitment should cease if the posterior mean lies outside the boundary.

The stopping boundary is obtained by solving what is known as an ‘optimal stopping problem’, using the techniques of dynamic programming [22, 23]. It is important to note that the solution to this problem uses information provided by the posterior distribution for the unknown value of $\mu _X$ and not just the expected value of the posterior distribution. That is, the expected benefit from stopping the trial uses a distribution which predicts the value of $\mu _X$ once remaining pipeline patients have been observed, and the expected value of recruiting an additional pair of patients (continuing the trial) weights optimal values for continuing the trial once that additional pair of patients has been recruited, using information derived from the posterior distribution. Full details of this process, and the so-called ‘Bellman equation’ which compares the expected values of stopping and continuing the trial, may be found in the discussion of Equations (6)–(8b) of [13].

In line with frequentist approaches to sequential trial design (see, for example, [24]), it is necessary to specify a maximum sample size for the clinical trial, represented here by the maximum number of pairwise allocations, $T_{\max }$, that can be recruited. In theory, $T_{\max }$ could be any value that the research team or funder chooses. There are a number of ways in which $T_{\max }$ could be chosen. For example, one method sets it to equal the sample size that would be set for a fixed sample size trial designed according to frequentist principles. This approach permits the trial to stop at, or before, the frequentist design’s target sample size. Alternatively, $T_{\max }$ could be set equal to the sample size which maximises the expected net benefit of sampling in a so-called ‘value of information’ calculation for a fixed sample size design [25]. Whatever method is chosen, the value-based sequential design stops the trial as soon as the estimated additional benefit of recruiting an extra pair of patients is estimated not to be worth the additional cost of doing so.

Under the value-based sequential model, the trial has three stages: during *Stage I*, patients are recruited and randomised to the two arms, but no accrual of cost-effectiveness data takes place because no patient has completed their follow-up period; during *Stage II*, values of INMB are observed sequentially and Eq. (2) are used to update the posterior distribution for $\mu _X$. After each observation of INMB, there is the option to randomise a further pair of patients to each arm of the trial, or stop recruitment. During *Stage III*, recruitment has stopped, but follow-up continues for the remaining pipeline patients. Once all patient outcomes have been observed and used to update the posterior mean for $\mu _X$, Stage III concludes and the decision about whether to adopt the new technology is made. Adoption of technology *N* is recommended if the total reward from adopting the technology exceeds any switching cost (that is, if $P \times \mu _{\tilde{n}} > I$, where $\tilde{n}$ is the total number of pairwise allocations made and $I \ge 0$ is the cost of switching from technology *S* to *N*).

**Fig. 1:**
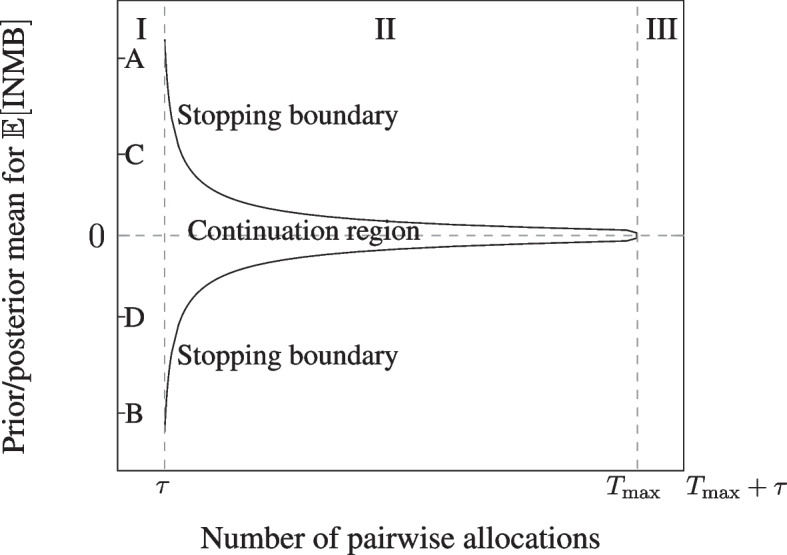
Stopping boundary for the value-based sequential model, showing the three stages of the trial (marked ‘I’, ‘II’ and ‘III’), the stopping boundary and the continuation region. [Source: adapted from [13, 18]]

Figure 1 shows how the policy works in practice for the case in which $I=0$. Consider first the region marked (Stage) ‘I’. Under the assumption that the prior mean, $\mu _0$, lies between the values indicated by points labelled ‘D’ and ‘C’ on the vertical axis, the sequential design is preferred. Recruitment takes place during Stage I and the first observation of INMB occurs once the first pair of patients have been followed up. The point marked $\tau$ in Fig. 1 is the delay to observation of outcomes, and is measured in terms of the number of pairwise allocations that are expected to have been made during the follow-up period for the first (and subsequent) observation(s) of INMB. During Stage II, as outcomes are observed, Eq. (2) are used to calculate the posterior mean and variance for $\mu _X$ in a series of interim analyses. If, at an interim analysis, the posterior mean lies within the area marked ‘Continuation region’ in Fig. 1, it is optimal to continue recruitment to the trial. The first time that an interim analysis shows that the posterior mean has crossed the upper or the lower part of the stopping boundary, it is optimal to halt recruitment and move to Stage III. During Stage III, cost and outcome data for the remaining patients in the pipeline are observed. Once all data from all pipeline patients have been observed and used to update the posterior mean for $\mu _X$, the adoption recommendation is made. If the posterior mean is greater than zero, technology *N* is recommended over technology *S*, otherwise it is not^2^.

There are two scenarios in which it is not optimal to run the sequential trial, defined because their expected rewards are higher than the expected reward of the sequential design. If the prior mean lies on, or between, the points marked ‘A’ and ‘C’ or ‘D and B’, it is optimal to run a fixed sample size trial where the optimal sample size is chosen so that the expected net benefit of sampling is maximised, according to established one-stage expected net benefit of sampling calculations (see, for example, [25]). We call such a trial design the ‘value-based one stage design’. If the prior mean is greater than ‘A’ or less then ‘B’, it is optimal to not run any trial and adopt *N* if $\mu _0 > A$ and adopt *S* if $\mu _0 < B$, for a reward equal to $P \mu _0$.

### The HERO trial

The HERO trial was a double-blind, randomised, clinical trial carried out in 13 primary and secondary care centres across England. It evaluated whether hydroxychloroquine is superior to placebo for the treatment of hand osteoarthritis (OA). Recruitment took place between 24 September 2012 and 27 May 2014, with follow-up completed on 29 June 2015. The study was funded by Arthritis Research UK (now Versus UK) and had a budget of £900,000.

For the clinical evaluation, follow-up of the primary endpoint took place at six months post-randomisation. For the economic evaluation it took place at 12 months post-randomisation. The trial protocol is published in [15] and results of the clinical evaluation are published in [16]. The original trial analyses/reporting were conducted according to CONSORT standards. Results of the within-trial economic evaluation are reported in [17]. Costs in the study were measured in UK £sterling, at 2015 prices.

The trial recruited 248 patients presenting with symptomatic pain and radiographic hand OA. Patients were randomised to receive either: (1) hydroxychloroquine in 200mg, 300mg or 400mg doses or (2) placebo. The primary clinical endpoint was average hand pain severity during the previous two weeks, measured on an eleven-point (0 to 10) numerical rating scale (NRS), at six months post-randomisation. Secondary endpoints, including quality of life, were also recorded. In particular, the trial used the EQ-5D-5L instrument to measure quality of life at baseline, 6 months and one year post-randomisation.

The economic evaluation consisted of a cost-utility analysis (estimating the cost per Quality Adjusted Life Year (QALY) at one year follow-up) and a cost-effectiveness analysis (estimating the cost per unit reduction in pain score). It was characterised by a considerable amount of missing data, particularly missing healthcare resource use data, a frequent problem in RCTs [26, 27]. The missing data problem is amplified when the summary measures used for analysis (e.g. total costs incurred during the follow-up period) are derived using repeated measurements of a large number of variables, as in the HERO trial’s economic evaluation. For example, the total cost associated with a given participant’s treatment and healthcare resource use during the follow-up period is missing if the participant is missing any one of the numerous variables that are used to derive this total.

The assumption that the missing cost and QALY data are ‘Missing Completely at Random’ (MCAR) is often less less plausible than the assumption that they are ‘Missing at Random’ (MAR) or ‘Missing not at Random’ (MNAR). In essence, MCAR means that the missing values are independent of both the observed and missing data, so that analysis which ignores them remains unbiased, albeit at the cost of precision. If the data are MAR, the missing values are not independent of observed data, potentially causing bias if this is ignored during analysis. In such a situation, multiple imputation and likelihood based methods can be used for valid, unbiased inference; see, amongst others, [26, 28–30]. Missing data are MNAR if the probability of missingness depends on the unobserved values themselves. The issue of MNAR outcome data in RCTs has received some interest recently [31, 32], but is beyond the scope of the current paper.

The base case economic analysis reported in [17] takes the perspective of the UK National Health Service and Personal Social Services and uses multiple imputation by chained equations under the assumption that the missing data are MAR [33–35]. Analysis of the clinical data found that hydroxychloroquine was not superior to placebo in terms of its effect on expected severity of pain at six months [16] and expected QALYs at one year [17]. The base case economic analysis found essentially no evidence that hydroxychloroquine is superior to placebo on cost-effectiveness grounds. Using a maximum willingness to pay for one QALY of £30,000, the estimate of expected incremental net monetary benefit of hydroxychloroquine compared to placebo was –£144.34 (95% confidence interval of (–£158.67, –£130.02)) and the probability that hydroxychloroquine is cost-effective was estimated to be 0.39 [17].

### Applying the Bayesian value-based sequential design to HERO

Referring to Eq. (1), we consider the new technology, *N*, to be hydroxychloroquine and the standard technology, *S*, to be placebo. Assuming a maximum willingness to pay for one QALY of £30,000, the incremental net monetary benefit for pairwise allocation *i* is:

3 $$\begin{aligned} \text {INMB}_i = {\pounds 30,000} (\text {E}_{i,\text {hyd}} - \text {E}_{i,\text {placebo}}) - (\text {C}_{i,\text {hyd}} - \text {C}_{i,\text {placebo}}). \end{aligned}$$

Positive values of $\text {INMB}_i$ indicate greater net benefit from hydroxychloroquine and negative values indicate greater net benefit from placebo.

Although the value-based sequential model can, in principle, operate in a fully sequential manner (that is, the posterior mean for $\mathbb {E}[\text {INMB}]$ during Stage II can be updated after each observed value of INMB and compared with the relevant Stage II stopping boundary), the analyses presented in this paper assume that the posterior mean is updated once every 10 pairwise allocations. This recognises the fact that continuous monitoring of the cost-effectiveness signal is unlikely to be feasible in most trials^3^. We assume that recruitment stops immediately following the first interim analysis that indicates the posterior mean for $\mathbb {E}[\text {INMB}]$ has crossed the stopping boundary.

Total costs and QALYs accruing during the follow-up period are derived in an identical manner to the original HERO economic analysis. For the purposes of this paper, point estimates of $\mathbb {E}[\text {INMB}]$ are obtained via simple comparisons of mean net monetary benefit between randomised groups, absent conditioning on any baseline covariates. This is sufficient to assess the performance of the value-based sequential model that is the focus of this paper, but is in contrast to the analysis reported in [17], which estimated $\mathbb {E}[\text {INMB}]$ using a seemingly unrelated regression model that conditioned on several baseline covariates.

Our analysis proceeded as follows. Firstly, we obtained the path of the posterior mean of $\mathbb {E}[\text {INMB}]$ using the actual trial data and assuming a time to follow-up equal to that used in the trial’s economic evaluation (12 months). Observations were ordered according to the date of randomisation, and we used multiple imputation to fill in missing values (refer to Handling missing data using multiple imputation section). We used the imputed datasets (generated using the entire sample) to obtain the estimate of the sampling standard deviation, $\sigma _X$. We used this estimate, together with estimates of other relevant parameter values (see Choice of parameter values section), to obtain the stopping boundary for the value-based sequential model. We then compared the path of the posterior mean to the stopping boundary to answer the question: ‘had the Bayesian value-based sequential model been used, when would the HERO trial have stopped?’ Next we considered the average performance of the value-based model by re-sampling from the HERO data and comparing these re-sampled paths of posterior mean with the relevant stopping boundary (Re-sampled data analysis section). In light of the fact that researchers have flexibility in setting the maximum sample size for the value-based sequential trial, $T_{\max }$, our main re-sampled data analyses set $T_{\max } = {124}$ and $T_{\max } = {248}$ pairwise allocations.

Finally, we carried out sensitivity analysis to investigate how robust our results were to: (1) increasing the maximum sample size to 1000 pairwise allocations and (2) reducing the time to follow-up of the cost-effectiveness data from 12 to 6 months.

#### Handling missing data using multiple imputation

The value-based sequential model assumes that the recruitment and follow-up of patients provides a series of independent and identically distributed observations of incremental net monetary benefit. If cost-effectiveness data are MAR or MNAR, then the observations of incremental net monetary benefit that are obtained using just the observed data on costs and utilities may not result in a representative sample from the population distribution of incremental net monetary benefit. As with any statistical analysis of incomplete data, the precise impact of the missing values will depend on the mechanisms that gave rise to them. These are, in general, not known. Hence the validity of any quantities obtained using the incomplete data, such as the posterior distribution for the expected value of incremental net monetary benefit, will generally rest on strong and largely unverifiable assumptions about the mechanisms that resulted in the missing data.

As noted in The HERO trial section, the HERO trial’s economic evaluation used multiple imputation by chained equations to address potential bias resulting from missing quality of life outcome and cost data, under the assumption that the missing values were MAR. We follow this approach in the analyses undertaken in this paper and use the same imputation model that was used for the base case analysis reported in [17]. Details of the variables included in the imputation model are given in Appendix Table 1. Assuming the imputation model encoded by this set of chained equations does a reasonable job of approximating the true joint model of the observed and incomplete cost-effectiveness data, the imputed datasets can be used to obtain unbiased observations of incremental net monetary benefit, which can then be used to update the posterior distribution. Clearly, this depends on unverifiable assumptions regarding the missing data mechanism. If for example, the missing data were truly MNAR, and particularly if the MNAR mechanisms differed by allocation, the observations of incremental net monetary benefit obtained from our imputed datasets may not be representative of the distribution that would have been obtained were the cost-effectiveness data complete. While a more comprehensive discussion of MNAR cost-effectiveness data is beyond the scope of the current paper, we note that recent work on sensitivity analyses using controlled multiple imputation - for example, [32, 36] - could be applied in the context of the value-based sequential model.

For each interim analysis, we firstly imputed missing cost and QALY data using all data available at the time of that interim analysis, generating five imputed datasets, as per [17]. We then obtained an estimate of $\mathbb {E}[\text {INMB}]$ for the most recent interim analysis by obtaining five estimates of $\mathbb {E}[\text {INMB}]$, one from each of the five imputations, for just the most recent block of pairwise allocations. We then combined these using Rubin’s rules [28, 37, 38]. These ‘by-block’ estimates were then used to obtain the values of the posterior mean and variance at each interim analysis using Eq. (2). It was not possible to obtain an estimate for the first interim analysis (that would have been based on 10 pairs) owing to data sparsity, which caused numerical difficulties for the chained equations algorithm used for the multiple imputation.

#### Re-sampled data analysis

We re-sampled observations with replacement from the HERO data, placed them into sequential blocks of 10 pairwise allocations based on a random order, and used the estimates of $\mathbb {E}[\text {INMB}]$ from these blocks to obtain the posterior mean of $\mathbb {E}[\text {INMB}]$, following the same approach to sequential multiple imputation as outlined in Handling missing data using multiple imputation section.

For the $T_{\max } = {248}$ analyses, 5000 paths were generated by drawing two re-samples of 124 pairwise allocations and placing them into a single dataset, with the 248 pairs then randomly sorted into sequential blocks of 10 pairwise allocations. We recognise that this approach uses the data twice, which places limitations on the statistical validity of conclusions drawn based on the re-sampled data for the $T_{\max } = {248}$ setting. However, in the absence of additional data, we feel that this is a reasonable approach to approximating the path of the posterior mean, had the original trial been permitted to run beyond its planned sample size of 248 patients. A further limitation of the re-sampling of participant level data described here is that it treats the observations as being independent, ignoring potential clustering of costs and health outcome data by centre. This is primarily because it is not possible to undertake re-sampling at the level of centre in the present study, because the centres recruited to the HERO trial differed substantially in terms of the number of patients they recruited. This implies that, were re-sampling to be undertaken by centre, there would be substantial fluctuations in the number of patients in each of the re-samples, would make it difficult to estimate sample sizes and research costs. While failure to properly account for dependence between observations obtained from patients recruited from the same cluster would compromise the frequentist properties of bootstrap standard errors and confidence intervals, we do not think this issue compromises our analyses. Again this is because the re-sampling undertaken in the present study was primarily a means of simulating some plausible paths of posterior mean of expected incremental incremental net monetary benefit with a weak signal, as opposed to being used for any formal frequentist inference. The re-sampled datasets for the $T_{\max } = {124}$ analyses were obtained by using just the first half of the randomly sorted re-sampled datasets generated for the $T_{\max } = {248}$ analysis. Appendix A provides further details.

To investigate the potential influence of increasing the maximum possible sample size of the HERO trial, we simulated trials with $T_{\max }$ set equal to the following values: 250, 500, 750, 1000, 1500, 2000, 2500, 3000, 4000 and 5000 pairwise allocations. We simulated 5000 replicates for each value of $T_{\max }$. In each case, $T_{\max }$ observations of incremental net monetary benefit were drawn from a Gaussian distribution with a mean of -£45 (as estimated using the multiply imputed HERO trial data), and a standard deviation of £7,615 (see Table 1). These simulated data were then used to obtain 5,000 paths of the posterior mean for the expected value of incremental net monetary benefit to compare with a value-based sequential model stopping boundary for the relevant value of $T_{\max }$.

#### Choice of parameter values

We used the parameter values reported in Table 1 to calculate the stopping boundary for the value-based sequential model. Here we discuss some of the main choices of parameter values. Full details about how each was chosen are presented in Appendix B.

We used the trial data to estimate the rate of accrual of patients and information on how the trial budget was spent to estimate the variable costs of research. For the valuation of the total benefit provided by the trial to the UK healthcare system, we set the maximum willingness to pay for one Quality Adjusted Life Year to £30,000. After reviewing literature on the prevalence and incidence of hand OA within the United Kingdom, we set the size of the population to benefit from the adoption decision, *P*, to 24,500 (equal to 2,450 patients per year for 10 years). Absent guidance about how fixed and variable costs in a clinical trial’s budget should be allocated, we assumed an even split between fixed and variable costs during the recruitment and follow-up periods. This gives an estimate of the cost of randomising a pair of patients of £1,650. We estimated $\sigma _X$ using multiply imputed data from all 124 patient pairs recruited in the trial.

We set the prior mean for the expected value of incremental net monetary benefit, $\mu _0$, to zero, reflecting the idea that, prior to the HERO trial, there was little evidence suggesting that hydroxychloroquine was more, or less, cost-effective than placebo. We set the prior variance, $\sigma ^2_0$, to give a low weight to the prior mean relative to the trial data, equivalent to an effective sample size of the prior of $n_0 = \sigma ^2_X / \sigma ^2_0 = {2}$ pairwise allocations. Our choice of prior distribution is intended to reflect the lack of cost-effectiveness information available to investigators prior to the trial taking place.

In line with the HERO trial’s economic analysis, we set the follow-up period for the cost-effectiveness data to be one year and we assumed a constant rate of recruitment to the trial that matched the average rate of accrual (124 pairs recruited over 611 days). Hence we assume that approximately $\tau = 74$ pairwise allocations were made by the time Stage II commences. This implies that, during Stage II, there are 74 pairs of patients in the so-called ‘pipeline’ of the trial. These are patients who have been randomised into the trial, but whose outcomes have yet to be observed. Hence, if the trial stops when an interim analysis has assessed outcome data for 30 patient pairs, the total sample size for the trial is 30 + 74 = 104 pairs, so 208 patients.

**Table 1:** Parameter values used to obtain the stopping boundary for the Bayesian value-based sequential model applied to the HERO trial

Parameter	Definition	Value	Source
	Estimated annual number of patients affected by the adoption decision	2,450	[39]
	Time horizon for the post-trial adoption population	10 years	Assumption
*P*	Number expected to benefit from the technology adoption decision	24,500	Defined from above parameters
$\sigma _X$	Standard deviation of incremental net monetary benefit in population	£7,615	Multiple imputation data sets
$n_{0}$	Effective sample size of the prior distribution for $\mathbb {E}[\text {INMB}]$	2 pairwise allocations	Assumption
$\mu _{0}$	Prior mean for $\mathbb {E}[\text {INMB}]$	0	Assumption
$\Delta$	Delay for observing EQ-5D-5L endpoint (in years)	1	[16]
	Estimated annual rate of recruitment to trial	74 pairwise allocations	[16]
$\tau$	Delay for observing EQ-5D-5L endpoint (in pairwise allocations)	74 pairwise allocations	Annual rate of recruitment
	Time horizon of trial	611 days	[16]
*I*	Fixed cost of adopting hydroxychloroquine	£0	HERO team advice
	Estimated spend on fixed costs prior to starting trial	£90,216	HERO trial’s accounts
	Estimated spend on fixed costs during trial	£204,581	HERO trial’s accounts
	Estimated spend on variable costs	£204,581	HERO trial’s accounts
	Estimated spend on fixed costs post follow-up	£336,042	HERO trial’s accounts
$c_{\text {fixed}}$	Total spend on fixed costs	£630,839	HERO trial’s accounts
	Total spend	£835,419	HERO trial’s accounts
*c*	Estimated cost per pairwise allocation	£1,650	HERO trial’s accounts
$\lambda$	Maximum willingness to pay for one QALY	£30,000	[20]

## Results

**Fig. 2:**
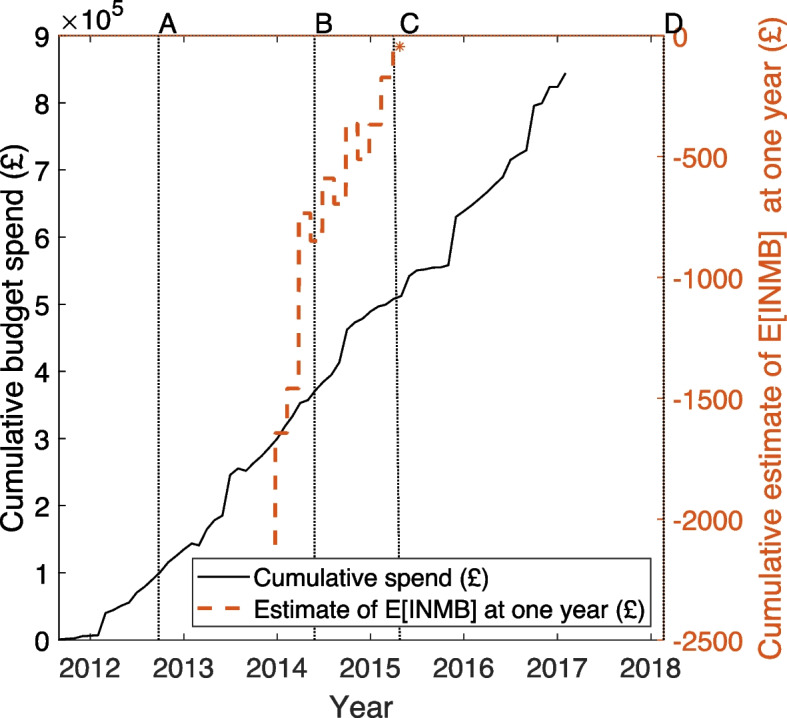
Estimate of cumulative budget spend (left axis, solid black line) and the point estimate of the expected value of incremental net monetary benefit (right axis, red dashed line) for the HERO trial. ‘A’ – recruitment starts; ‘B’ – recruitment finishes; ‘C’ – one year follow-up finishes; ‘D’ – principal publication of the clinical analysis [16]. The cost-effectiveness data are presented in column 5 of Appendix Table 2

First, we consider the HERO trial’s research expenditure and cost-effectiveness signal over time. The black continuous line in Fig. 2 (left axis scale) plots the cumulative spend of its research budget, using data from the financial accounts. Cumulative spend includes all costs recorded in the financial accounts, for whatever reason. Also plotted as a red dashed line on the right axis scale is the estimate of $\mathbb {E}[\text {INMB}]$ at one year as evidence from the trial accumulated. These sequential point estimates are based on the multiply imputed data. The plotted values are given in column (5) of Appendix Table 2, with key milestones in the project marked as follows: ‘A’ (recruitment starts); ‘B’ (recruitment finishes); ‘C’ (one year follow-up finishes); ‘D’ (publication of [16], presenting the results of the clinical evaluation).

Figure 2 shows that, during follow-up, the estimate of $\mathbb {E}[\text {INMB}]$ was never greater than zero, meaning that there was never evidence that hydroxychloroquine was cost-effective. The first estimate, based on cost and outcome data from the first 20 pairs of patients allocated, is equal to –£2172. By the end of follow-up, the estimate had risen to –£45, with a 95% confidence interval of (-£1387 to £1296). This implies that the trial provides little evidence that one technology is superior to the other on cost-effectiveness grounds, which we take to be an ‘equivocal’ cost-effectiveness signal.

Comparison of the spend and cost-effectiveness profiles provides insight into how much, if any, of the research budget might have been saved had the trial been allowed to stop recruitment early: approximately one third of the trial’s budget had been spent by the time that one year follow-up commenced and just under 60% had been spent by the time it had finished (‘C’). Crucially, around 45% had been spent by the time recruitment finished (‘B’). This means that only about 12-15% of the trial’s expenditure occurred between the beginning of the one year follow-up period and the end of participant recruitment.

**Fig. 3:**
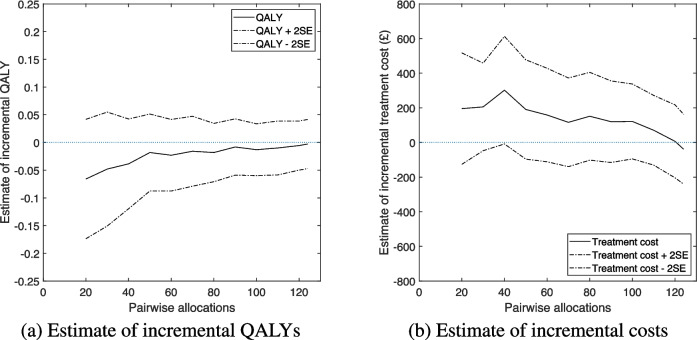
Estimate of expected incremental QALYs and treatment costs at one year as evidence accumulated, together with limits at ± two standard errors, using imputed data. For the expected incremental QALYs values above zero suggest hydroxychloroquine to be superior to placebo and for the expected incremental treatment cost values above zero suggest hydroxychloroquine to be more expensive than placebo. The data series for **a** is presented in column 3 of Appendix Table 2. The data series for **b** is presented in column 4

Figure 3 breaks down the sequential point estimates of $\mathbb {E}[\text {INMB}]$ at one year that are plotted in Fig. 2 into estimates of expected incremental QALYs (Fig. 3a) and expected incremental treatment costs (Fig. 3b) at one year. Limits showing plus and minus two standard errors are also shown, to provide some indication of the uncertainty surrounding the estimates. Values above zero show hydroxychloroquine to be more effective (Fig. 3a) / more costly (Fig. 3b). The plots show that hydroxychloroquine was estimated to be less effective than placebo throughout the follow-up period, although the final estimate of incremental QALYs is very close to zero. Figure 3b shows that treatment with hydroxychloroquine was estimated to be more expensive than placebo throughout the follow-up period, except at the very end, when it was estimated to be £39 cheaper. These plots explain the equivocal estimate of cost-effectiveness that is shown in Fig. 2.

**Fig. 4:**
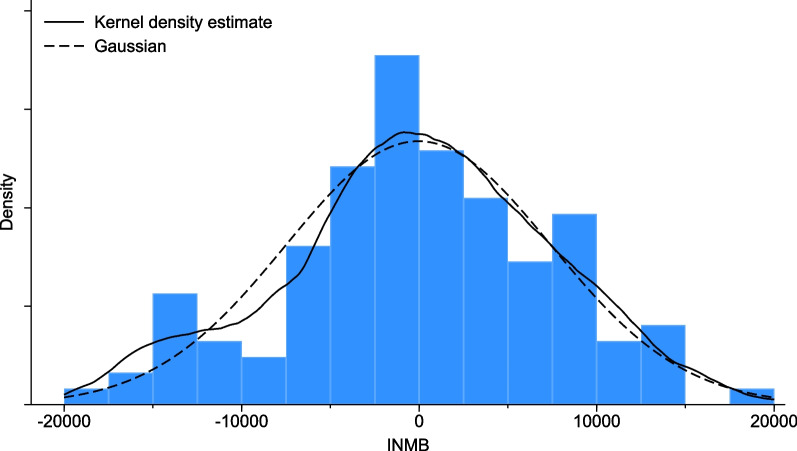
Histogram showing the distribution of the paired observations (paired according to their order of arrival in the data set) of incremental net monetary benefit for the HERO trial. The solid black line shows a kernel density estimate based on an Epanechnikov kernel, and the dashed black line shows a Gaussian distribution with mean and variance equal to the sample mean and variance of the 124 observations of pairwise INMB

Finally, a plot of the pairwise INMB data is presented in Fig. 4, where observations have been paired according to their order of arrival in the data set. The histogram is superimposed with a kernel density estimator and a Gaussian distribution with the same mean and variance as the sample mean and variance of the observations on INMB. Our tests for normality of the INMB data did not reject the null hypothesis of normality at the 5% significance level. The Shapiro-Wilk test, Shapiro-Francia test and Skewness-Kurtosis test gave p values of 0.439, 0.406 and 0.680, respectively.

### Running the HERO trial as a value-based sequential design

**Fig. 5:**
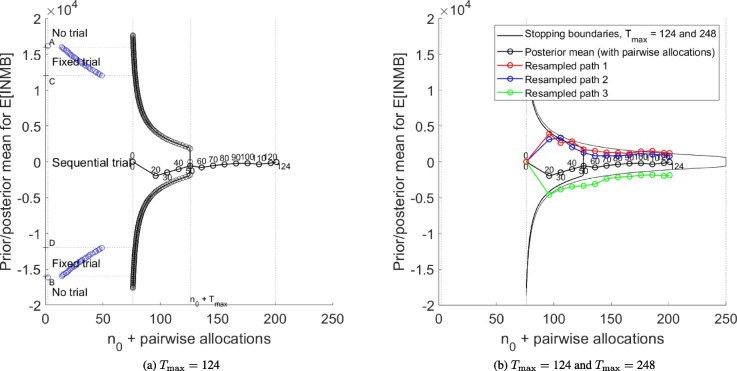
Stopping boundary and paths for the posterior mean for the HERO trial for: **a** the case of $T_{\max } = {124}$ and **b** $T_{\max } = {124}$ and $T_{\max } = 248$, together with three **resampled** paths

Figure 5a presents the stopping boundary for the value-based sequential model applied to the HERO trial when the maximum sample size is set equal to the trial’s actual sample size (124 pairwise allocations). The Stage II stopping boundary is marked in black, using unnumbered, circled points linked by a continuous line. Also marked are the letters ‘A’ to ‘D’, showing the ranges of the prior mean for which no trial, a value-based one stage design and the value-based sequential design are optimal (refer to Fig. 1). Where the value-based one-stage design is optimal, a range of optimal sample sizes for that design is indicated by blue circles. Figure 5a shows that, under the chosen parameter values (refer to Table 1), the value-based sequential design is optimal, from the perspective of maximising overall expected net benefit to the health care system, if the absolute value of the prior mean for $\mathbb {E}[\text {INMB}]$ is less than about £12,000 (points C and D). It also shows that no trial is optimal if the absolute value of the prior mean for $\mathbb {E}[\text {INMB}]$ is greater than about £16,000, with immediate adoption of hydroxychloroquine recommended only if the prior mean exceeds £16,000.

As noted in the Choice of parameter values section, we assume a prior mean for the expected value of incremental net monetary benefit that is equal to £0. Since this value lies between the points C and D, the sequential design is optimal. Figure 5a also shows the path of the posterior mean for the expected value of incremental net monetary benefit, obtained using the multiply imputed HERO trial data, assuming that interim analyses take place every ten pairwise allocations (with the exception of the first interim analysis, which takes place at 20 pairwise allocations for the reason stated in Handling missing data using multiple imputation). The path remains in the continuation region throughout Stage II, showing that, under the value-based sequential design, recruitment would have continued until the sample size of the actual trial, 124 pairwise allocations, had been reached, and would have resulted in a final estimate of the posterior mean equal to approximately –£30 (hydroxychloroquine not cost-effective) and a technology adoption recommendation consistent with the results of the original trial, that is, that hydroxychloroquine should not be adopted.

This part of our application shows that, had the HERO trial been run according to the value-based sequential trial model, with a switching cost, *I*, assumed equal to zero, it would not have stopped before reaching the maximum planned sample size, and therefore the sequential design would not have saved any of the trial’s research budget. The principal reason for this is the relatively weak cost-effectiveness signal in the trial. However, an additional factor is the relatively small number of interim analyses (three) that occurred during Stage II of the trial, which offer limited scope for early stopping, and therefore limited scope for the sequential design to deliver increase value via reduced research expenditure.

### Re-sampled data analysis

Figure 5b shows the same Stage II stopping boundary and path of the posterior mean that are plotted in Figure 5a, together with the stopping boundary for $T_{\max } =248$ and three re-sampled paths for the posterior mean generated according to the procedure described in Re-sampled data analysis. These paths show three scenarios in which the value-based sequential design would cease recruitment before reaching a maximum sample size of 124 pairwise allocations. For example, ‘Re-sampled path 1’ first crosses the Stage II stopping boundary at the third interim analysis, informed by outcome data from the first 40 pairwise allocations, at which point recruitment stops, having recruited a total of 114 pairs of patients – the 40 that contributed to the interim analysis, plus the 74 ‘pipeline’ pairs. The posterior mean upon conclusion of follow-up is positive and so favours adoption of hydroxychloroquine. Similarly, ‘Re-sampled path 3’ crosses the stopping boundary at the first interim analysis, after outcomes for 20 pairwise allocations have been observed, so that 94 pairwise allocations have been recruited to the trial. However, for this path, the final estimate of $\mathbb {E}[\text {INMB}]$ is negative and so favours placebo.

As described in Re-sampled data analysis, in our main analysis we obtained 5000 paths for two different trial scenarios, $T_{\max } = 124$ and $T_{\max } = 248$. We obtained summary statistics regarding the final estimate of the posterior mean of $\mathbb {E}[\text {INMB}]$ and the number of pairs randomised, and compared them with fixed length designs equal to the chosen values of $T_{\max }$. Table 2 presents summary statistics for the two scenarios. The proportions of re-sampled paths that conclude that hydroxychloroquine is cost-effective under each of the designs are presented in Table 3.

**Table 2:** Re-sampled data analysis: comparison of the performance of the value-based sequential model designs with fixed sample size designs with maximum sample sizes of 124 and 248 pairwise allocations

	Average	Standard deviation	Minimum	Maximum
**Maximum sample size ** $\varvec{=}\ \varvec{124}$ **pairwise allocations**				
*HERO trial (original fixed sample size design)*				
Posterior mean for $\mathbb {E}[\text {INMB}]$ (£)	-91.47	647.51	-2559.69	2538.94
*Value-based sequential model*				
Posterior mean for $\mathbb {E}[\text {INMB}]$ (£)	-92.85	654.24	-2817.26	2538.94
Sample size (pairwise allocations)	123.56	2.93	94	124
**Maximum sample size ** $\varvec{=}\ \varvec{248}$ **pairwise allocations**				
*Fixed length trial*				
Posterior mean for $\mathbb {E}[\text {INMB}]$ (£)	-87.78	474.12	-1683.60	1804.17
*Value-based sequential model*				
Posterior mean for $\mathbb {E}[\text {INMB}]$ (£)	-101.07	543.69	-2559.69	2466.24
Sample size (pairwise allocations)	236.91	27.77	94	248

**Table 3:** The proportion of re-sampled paths which suggest that hydroxychloroquine is cost-effective, for the designs summarised in Table 2

	Final decision	
	Hydroxychloroquine not cost-effective	Hydroxychloroquine cost-effective
**Maximum sample size ** $\varvec{=}\ \varvec{124}$ **pairwise allocations**		
*HERO trial (original fixed sample size design)*	0.552	0.448
*Value-based sequential model*	0.552	0.448
**Maximum sample size ** $\varvec{=}\ \varvec{248}$ **pairwise allocations**		
*Fixed length trial*	0.570	0.430
*Value-based sequential model*	0.570	0.430

When the maximum sample size of the value-based sequential model is set to 124 pairwise allocations, only around 3% of the re-sampled paths cease recruitment before the maximum sample size. As alluded to in the Running the HERO trial as a value-based sequential design section, this is due to the equivocal cost-effectiveness signal in the trial data, combined with the small number of interim analyses that can take place during Stage II. As a result, the final estimates of the posterior mean of $\mathbb {E}[\text {INMB}]$, and the expected sample size and the proportion of re-sampled paths that conclude that hydroxychloroquine is cost-effective are very similar to those of the fixed sample size design. Under the assumed cost per pairwise allocation of £1,650 (see Table 1), the small reduction in expected sample size under the value-based sequential approach translates to an estimated cost-saving for the trial of around £700 in total, less than 0.1% of the HERO trial’s budget.

When the maximum sample size is set to 248 pairwise allocations, about 22% of the re-sampled paths cease recruitment before the maximum sample size is reached. This is driven by the increased length of Stage II, which now permits 16 interim analyses. However, the sample sizes for these ‘early-stopping’ paths are generally quite close to the maximum number of pairwise allocations permitted in the trial, again due to the equivocal cost-effectiveness signal. As a result, the expected sample size is only around 4.5% smaller for the value-based sequential design than it is for the fixed length design. Applying the same assumptions as before, this translates into an estimated cost saving of around £18,000 (=(248-237) $\times$ £1650) over the fixed design. Despite there being more paths stopping early under this version of the value-based sequential model, the final estimates of $\mathbb {E}[\text {INMB}]$ for the fixed and sequential designs are again similar. Finally, the proportions of paths favouring hydroxychloroquine from the cost-effectiveness perspective, equal to 0.430, are essentially identical across the two $T_{\max } = 248$ designs that we consider, albeit being slightly lower than the proportions observed for the $T_{\max } = {124}$ designs (0.448).

To summarise, the qualitative message from the application of the value-based sequential model to the HERO trial is that, contrary to the findings reported in [18] for the ProFHER trial, there is little prospect of stopping earlier than the planned maximum sample size of the trial, and therefore little prospect of saving research monies, regardless of whether the maximum sample size is set at 124 or 248 pairwise allocations. This is primarily due to the equivocal evidence concerning cost-effectiveness in the HERO trial, with a secondary reason being the limited number of Stage II interim analyses that are feasible, given the choices of $T_{\max }$.

### Sensitivity analyses

To test how our main result is affected by alternative specifications of the design, we undertook two additional analyses. The first of these analyses increased the trial’s maximum possible sample size, examining the operating characteristics of the value-based sequential model for $T_{\max }$ equal to the following values: 250, 500, 750, 1000, 1500, 2000, 2500, 3000, 4000 and 5000 pairwise allocations. The second analysis reduced the delay to observing cost-effectiveness outcomes (from 12 months to 6 months). Both of these specifications increase the number of interim analyses during Stage II. Further methodological details are contained in Appendix D.

**Fig. 6:**
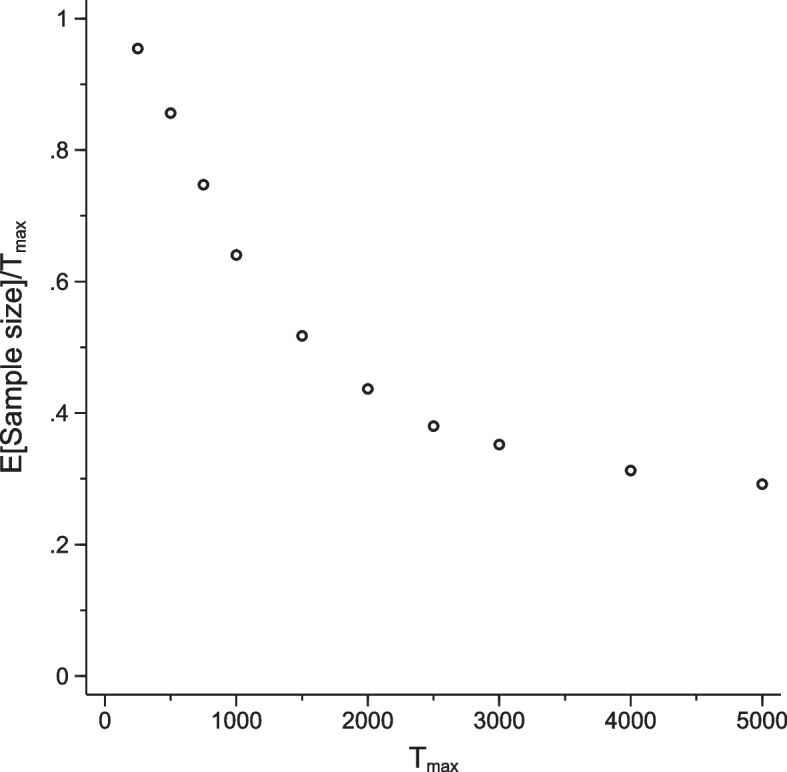
The ratio of expected sample size to $T_{\max }$ as a function of $T_{\max }$ for the HERO trial

The impact of increasing $T_{\max }$ on expected sample size is plotted in Fig. 6. This plot shows the ratio of the expected sample size of the value-based sequential trial (across the 5000 replicates) to $T_{\max }$ as a function of $T_{\max }$. We found that, while expected sample size increases as $T_{\max }$ increase, it appears to do so at a diminishing rate. For example, when $T_{\max } = {250}$ the average sample size across the 5000 simulated paths was 239 (96% of $T_{\max }$), roughly aligning with the re-sampled data analyses undertaken for $T_{\max } = {248}$ (see Re-sampled data analysis section). For $T_{\max } = {1000}$ it was 640 (64% of $T_{\max }$) and by $T_{\max } = {5000}$ it was 1460 (29% of $T_{\max }$). These results suggest that even in the context of a very weak cost-effectiveness signal, the value-based sequential model can deliver substantial reductions in expected sample size and variable costs, compared to an equivalent fixed sample size design. However, they also suggest that large values of $T_{\max }$ may be required to realise important reductions in these quantities. For example, $T_{\max } = {1000}$ pairwise allocations is around eight times the sample size of the original trial, and the expected sample size of 640 pairwise allocations is more than a five-fold increase in the sample size of the original trial, equating to an increase in research costs of approximately £851,400 (assuming a cost per pairwise allocation of £1,650). Further, around 27% of the simulated paths continued to 1000 pairwise allocations. Indeed, the proportion of simulated paths reaching $T_{\max }$ dropped rapidly from around 77% when $T_{\max } = {250}$ to around 20% for $T_{\max } = {2000}$, but changed little thereafter with around 19% of simulated paths running to $T_{\max }$ for all values of $T_{\max }$ larger than 2000. Finally, we note that despite the clear impact of the choice of $T_{\max }$ on expected sample size, the final estimates of expected incremental net monetary benefit and the proportion of paths concluding in favour of hydroxychloroquine varied little with changes in $T_{\max }$. A full set of operating characteristics are shown in Appendix Tables 3 and 4 for the $T_{\max } = {1000}$ scenario.

On the one hand, these results suggest that, in the context of an equivocal cost-effectiveness signal, the value-based sequential model can provide a meaningful reduction in expected research expenditure if given sufficient opportunity to do so. On the other hand, it is perhaps unrealistic to think that a healthcare system would set a maximum sample size so large, relative to the planned sample size of the frequentist design. We therefore do not consider the substantial reductions in expected sample size evident for $T_{\max } = {1000}$ and above to alter materially the conclusions of our main analyses, at least in the context of the HERO trial.

Appendix Table 3 shows that halving the time to follow-up for measuring the cost-effectiveness outcomes, by setting a six month time horizon instead of a 12 month horizon, has relatively little impact on the results. For the trial with $T_{\max } = {124}$, the expected sample size for the value-based sequential design was 120 pairwise allocations. When $T_{\max } = {248}$, the value-based sequential design showed a modest reduction in expected sample size of around 30 pairwise allocations compared with the fixed length design. In both cases the additional value delivered to the healthcare system via reduced costs of research is small.

## Discussion

The analysis reported in this paper represents only the second published application of the Bayesian value-based sequential model of [13, 14] to data from a clinical trial. It is the first application to investigate the behaviour of the model in the presence of an equivocal cost-effectiveness signal, and also the first to use multiple imputation to address the problem of missing cost-effectiveness data.

Table 4 compares some of the principal results reported in the Results section with those from the application of the value-based sequential model to data from the ProFHER trial [18]. Column (2) reports the final estimate of $\mathbb {E}[\text {INMB}]$ based on the original trial data, showing that the cost-effectiveness signal was much stronger in the ProFHER trial than in the HERO trial. Columns (4) to (7) show the actual and percentage changes in the sample size and budget when $T_{\max }$ is equal to the actual sample size of the trials (124 pairwise allocations for HERO, 125 for ProFHER) and with a sample size equal to double that number. For columns (6) and (7), which report the results for the re-sampled data, the figures are based on the expected values. Column (8) reports the percentage of re-sampled paths that report a result consistent with the trial’s recommendation (that surgery is not cost-effective (ProFHER); that hydroxychloroquine is not cost-effective (HERO)).

The table shows that the value-based sequential model offers non-negligible savings in sample size and budget in the ProFHER application (equal to 14% for the trial’s sample size and 5% of the budget, with averages from the re-sampled data estimated to be 42%/38% and 14%/13%, respectively), but not the HERO application. This is principally due to the strong evidence suggesting that surgery is not cost-effective in the ProFHER trial (the final estimate of the expected value of incremental net monetary benefit is -£1758), a result not reflected in the HERO trial, where the equivalent figure is -£45.

**Table 4:** Performance of some of the principal operating characteristics in the ProFHER and HERO applications of the Bayesian value-based sequential model

(1)	(2)	(3)	(4)	(5)	(6)	(7)	(8)
	Estimate of $\mathbb {E}\varvec{[}\textbf{INMB}\varvec{]}$(£) (95% CI)	$\varvec{T}_{\textbf{max}}$	Original trial data		Re-sampled data		% paths consistent with trial recommendation
			Sample size	Budget % change	Sample size Mean (SD)	Budget % change	
ProFHER	-1758 (-2389, -1126)	125^a^	107	-5	73 (19)	-14	91
		250	**-**	**-**	77 (27)	-13	92
HERO	-45 (-1387, 1296)	124^a^	124	**0**	124 (3)	0	55
		248	**-**	**-**	237 (28)	+22	57

^a^The trial’s actual sample size, measured in pairwise allocations

The absence of material reductions in expected sample size or costs in the HERO application should not be taken to be a negative result. Early termination of recruitment would generally not be indicated, or indeed desirable, in such a scenario. The absence of evidence of early stopping indicates that the expected benefits from continuing to learn about the comparative cost-effectiveness of the two technologies for the 24,500 patients who will be impacted by the adoption decision is, in general, greater than the expected benefits of stopping recruitment during Stage II.

Our results also show that the impact of the equivocal cost-effectiveness signal on the expected cost-savings delivered by the value-based sequential model is affected by the duration of Stage II, as well as the proportion of variable costs committed by the time Stage II starts. This is particularly evident for the scenario which sets the maximum sample size of the sequential design, $T_{\max }$, to be the sample size chosen for the HERO trial (124 pairwise allocations), with a time to follow-up of cost-effectiveness data equal to one year. In this scenario, 60% of patients are randomised into the trial before Stage II starts and only three interim analyses occur prior to $T_{\max }$ being reached. Hence, even in the presence of a stronger cost-effectiveness signal, the cost-saving offered by the value-based sequential model is likely to be small. In contrast, for the application to the ProFHER trial, only 38% of the maximum sample size was committed prior to the start of Stage II and seven, not three, interim analyses could be undertaken during Stage II. This relationship between the recruitment horizon and time to follow-up is consistent with what has already been observed in the literature [40].

It is important to note that there is no requirement to set $T_{\max }$ to the sample size chosen for the conventional frequentist design. It could be set to a value that is considerably greater than that, such as the $T_{\max } = {248}$ scenario we have considered. Or it could be set to the sample size that would maximise the expected net benefit of sampling, using the Bayesian one-stage trial design principles, which would permit comparison of expected values of the one stage and the value-based designs to be made^4^. Such an increase could be advantageous in terms of maximising the overall net-benefit delivered by the value-based sequential model. Of course, if a strong cost-effectiveness signal emerges during Stage II, the value-based sequential model is likely to terminate recruitment well before $T_{\max }$ is reached, as is shown in the ProFHER application. It would also be interesting to explore in more detail the implications of deploying the model in very large trials, buiding on the analysis that is reported in the Sensitivity analyses section.

One further difference between the ProFHER and HERO applications that is evident from Table 4 concerns the proportion of re-sampled paths that result in a decision consistent with the results of the original trial. For the ProFHER trial, more than 90% of the paths show that surgery is not cost-effective in the UK setting. For the HERO trial, only around 55% of paths conclude in favour of placebo. This is again due to the difference in the strength of the cost-effectiveness signal between the two studies, but it is also a consequence of the differences in inferential and/or decision-making criteria that are adopted by the value-based sequential model and the original frequentist methods, particularly with regards to the strength of evidence that is required to induce a switch to a new health technology. If the one-time switching cost of adopting the new technology (i.e. hydroxychloroquine in the HERO trial) is assumed to be £0, then under the value-based sequential model, the new treatment should be adopted if and only if the final estimate of the posterior mean of $\mathbb {E}[\text {INMB}]$ exceeds £0. Given the equivocal cost-effectiveness signal in the HERO data, a reasonably large proportion of the re-sampled paths – approximately 45% – conclude with a posterior mean that is slightly greater than £0. This is in contrast to the frequentist approach (see [17]), for which hydroxychloroquine would only have been recommended for adoption if the data provided sufficient information to refute the null hypothesis of no difference in a direction favouring hydroxychloroquine. A discussion of the advantages and disadvantages of different systems of inference and decision-making is beyond the scope of this paper. However, the asymmetry and conservatism of the frequentist approach – which would likely be desirable if a given technology is expected to impose important costs to the health system – can be incorporated into the value-based sequential model in an explicit and readily interpretable way, via the inclusion of the non-zero switching cost $I>0$ in the derivation of the optimal policy.

The quantitative findings that we report in the Results section are dependent on the precise values of the various parameters what we have chosen for our application, including the size and timing of interim analyses. However, the qualitative results, and the contrast between the HERO and ProFHER results, are likely to be relatively insensitive to any reasonable choice of parameters, owing to the nature of the cost-effectiveness signals from the two trials. That said, a limitation of our analysis is that all choices of parameter values were fully retrospective, and in some cases, were based on the observed trial data and records of actual trial expenditure. In practice, the unknown parameter values required for the value-based sequential model would need to be specified during trial set-up. Obtaining accurate estimates of some of these parameters prospectively could be challenging, although we note that the issue of specifying prospective estimates of unknown design parameters is by no means unique to the value-based sequential approach.

As an example, consider estimation of the delay to observing outcomes in terms of pairwise allocations ($\tau$). This requires an accurate estimate of the expected rate of patient recruitment during the trial, as well as the time horizon for follow-up. Although trial teams generally specify target recruitment figures during trial set-up, observed rates of accrual can differ considerably from those that are anticipated. While small departures from the anticipated rate of accrual may not be a major issue, large deviations could compromise the validity of the Stage II stopping boundary because the number of pipeline patients may differ considerably from the planned number. One way that this could be addressed in practice is by using an internal pilot phase to assess the rate of accrual, and modify the Stage II stopping boundary accordingly.

A second example concerns the number of patients, *P*, affected by the technology adoption decision. This depends on both the incidence of the condition and the time horizon over which the adoption decision will apply. While, from a value-based perspective, it is clear that these parameters are a prerequisite to informed and rational decision making, in practice there is likely to be uncertainty regarding both incidence and time horizon. Further work could explore the practicalities of eliciting prospective estimates of these parameters, as well as the potential impact of discrepancies between such estimates and their true values.

A final example concerns the cost per pairwise allocation, *c*. Our estimate of this parameter was derived under the assumption of an even split between fixed and variable costs during the recruitment and follow-up periods, a strong and probably overly simplified assumption. We believe our results concerning sample size and resource savings in the Results section are unlikely to be materially affected by small-to-moderate changes in this input, at least for $T_{\max } = {124}$ or 248. However in other scenarios, accurate estimation of the cost per pairwise allocation could be of great importance in terms of its impact on both the optimal policy and any cost-savings that might be obtained by stopping recruitment early under the sequential design. While there is some literature on costs per patient in the commercial context (for example, [41]), there is little published literature providing figures for non-commercial clinical trials (such as the HERO trial). Published data concerning expected costs per patient in the non-commercial context would likely be of considerable value to any future work investigating the potential economic benefits of sequential clinical trials, whether they take a value-based perspective or otherwise.

The analyses reported in this paper assumed a fixed, known value for the sampling standard deviation of pairwise observations of incremental net monetary benefit, $\sigma _X$. We based this estimate on the observed data, but in practice, a reasonable estimate of $\sigma _X$ is required prospectively in order to derive the optimal policy. It is worth noting that accurate specification of variance/nuisance parameters prior to a trial’s commencement is also necessary for many other approaches to trial design, whether they be frequentist or Bayesian. Furthermore, the assumption that the sampling standard deviation, $\sigma _X$, is known can be relaxed so that the prior-posterior distributions of both the expected value of incremental net monetary benefit and the variance of incremental net monetary benefit are updated as outcomes are observed (see Section 4 of [13]). Finally, although our tests of normality of the data for incremental net monetary benefit were not rejected in the HERO trial, the general question of the performance of the model when data are not normal is an interesting topic for future research.

A further area for future research effort is to consider the additional costs of designing and running a trial according to the value-based sequential model. It is plausible that, although increasing the number of interim analyses introduces additional flexibility and is therefore likely to deliver better value, the additional costs arising from frequent monitoring could outweigh this increase in expected net benefit. Future work could consider how to estimate the additional costs of running a trial according to the value-based sequential model (possibly following similar methods to those used in [12]), and the extent to which this impacts the expected net benefit of this approach over some comparable designs.

We also did not explore alternative approaches to incorporating multiple imputation into the sequential analyses that were undertaken as part of our application of the value-based sequential model, or the potential impacts of leveraging informative baseline covariates when obtaining estimates of $\mathbb {E}[\text {INMB}]$. While the qualitative results for HERO are unlikely to be particularly sensitive to either aspect, there might be alternative trial settings where these analytical choices matter more. Future work could explore different methods of incorporating both multiple imputation and more sophisticated model-based estimation of $\mathbb {E}[\text {INMB}]$ into the value-based sequential approach, and their advantages and disadvantages.

Two final matters are worthy of note. Firstly, we have focused exclusively on applying the value-based sequential model in the context of two-arm individually randomised trials. This is motivated by the theory underlying the value-based sequential model of [13], which focused on this setting. However, there are potential avenues for theoretical developments to extend the value-based sequential framework to handle hierarchical data as, for example, are encountered in cluster-randomised trials [42–44]. Secondly, there exist alternative metrics to evaluate the value-based sequential design, using metrics from the Bayesian value of information literature (see [25] and related literature). These were deemed beyond the scope of this article, but are included in Appendix E for the interested reader.

## Conclusions

We have investigated the implementation of the Bayesian value-based sequential model proposed by [13, 14] in the context of the HERO trial’s equivocal cost-effectiveness signal, and illustrated how multiple imputation might be used to address missing data within this framework. Considered alongside the findings from the ProFHER application, our results suggest that, in the presence of an unambiguous cost-effectiveness signal, such as in the ProFHER trial, the value-based sequential model can produce material reductions in expected sample size and research costs, but that this is not the case when the signal is equivocal, such as in the HERO trial. This work helps build a more complete picture of the behaviour of the value-based sequential model under different scenarios, which can help inform any future prospective application of this approach alongside existing trial designs and decision making criteria.

### Supplementary Information

Supplementary Material 1.

## Data Availability

Analysis of the original trial data used Stata 13 [35] and the multiple imputation used the ice command of [33, 34]. Calculation of the stopping boundaries used the Matlab code that is available from https://github.com/sechick/htadelay and used Matlab R2022a [45]. Re-sampling and multiple imputation were undertaken using Stata 17 [46] with the generated paths of posterior mean of expected incremental net monetary benefit being analysed using Matlab R2022a (with replication using Stata 17).

## Abbreviations

CAT: Costing of adaptive trials project
ENACT: Economics of adaptive clinical trials project
HERO: Hydroxychloriquine effectiveness in reducing symptoms of hand osteoarthritis
INMB: Incremental net monetary benefit
MAR: Missing at random
MCAR: Missing Completely at random
MNAR: Missing not at random
NIHR: National institute for health and care research
NRS: Numerical rating scale
OA: Osteoarthritis
ProFHER: PROximal fracture of the humerus: evaluation by randomisation
QALY: Quality adjusted life year

## Footnotes

1 The principal publication from the ENACT project is [11]. A principal publication from the CAT project is [12] and the project’s website is https://www.newcastle-biostatistics.com/methodology_research/adaptive_designs/

2 The Stage III that is labelled in Fig. 1 refers to a trial which runs to the maximum sample size, $T_{\max }$. Stage III starts earlier if Stage II finishes before reaching $T_{\max }$.

3 In principle, interim analyses could take place as frequently, or as infrequently, as desired. We have chosen to hold an interim analysis every ten pairwise allocations because we believe it strikes a reasonable balance between continuous data monitoring during the trial – we believe this to be unrealistic – and only monitoring the data once during Stage II – which, we believe, minimises the sequential benefits which could be provided by the model.

4 Although not the central focus of this paper, we note that such comparisons are reported in the HERO trial in [11].
