# Supplementary material for: The performance of a Bayesian value-based sequential clinical trial design in the presence of an equivocal cost-effectiveness signal: evidence from the HERO trial
Source: BMC Med Res Methodol. 2024 Jul 19;24:155. doi: 10.1186/s12874-024-02248-9 (PMC11264712; doi:10.1186/s12874-024-02248-9)
Supplement: Supplementary file 1 — Supplementary Material 1. [file 12874_2024_2248_MOESM1_ESM.pdf]

Appendix A Procedure used to generate paths for the posterior mean

This section describes the approach taken to generating the re-sampled paths for the posterior mean of the expected value of incremental net monetary benefit that are presented in Figure 5b of the main manuscript. We generated 5000 re-sampled trials assuming a maximum sample size of 248 pairwise allocations and then truncated the simulated paths of the posterior mean to obtain the paths for the case of $T_{\max} = 124$. The following steps describe the procedure used to generate one re-sampled path for the trial with 248 pairwise allocations.

1. Draw (with replacement) 248 observations from each allocation stratum (248 from hydroxychloroquine, 248 from placebo) from the original HERO dataset.
2. Randomly sort this resample and place it into 25 blocks of 10 pairwise allocations (24 blocks of 10 pairwise allocations, plus one block of 8 pairwise allocations).
3. For $i = 2, \ldots, 25$ blocks, impute missing values as follows:
   (a) use all patients in blocks $1, \ldots, i$ (e.g. for block 8, use the 80 hydroxychloroquine patients and the 80 placebo patients in blocks 1–8);
   (b) if all patients have a value of either 0 or missing for a given variable, then replace the missing values with 0 (this step is required primarily for some of the cost variables in the first few blocks);
   (c) identify variables with missing values that will lead to severe collinearity in the univariate models fitted as part of the multiple imputation by chained equations (MICE) procedure [1, 2] and exclude them from that procedure (this generally applied to cost variables in early blocks, where the majority of values are either 0 or missing, with only a small proportion of non-zero, non-missing values);
   (d) impute incomplete variables using MICE [1, 2], generating five datasets where these variables are complete. The variables included in the imputation model are described in Appendix Table 1;
   (e) impute missing values in variables excluded from the MICE procedure (if any) using hotdeck multiple imputation (i.e. missing values are imputed with observed values in proportions that match the observed data, and imputed values can vary within individual between imputed datasets).
4. For blocks 2,. . . ,25, calculate a point estimate of expected value of incremental net monetary benefit for each of the five imputed datasets and combine these point estimates by

taking their mean (as per Rubin's rules [3, 4, 5]), resulting in point estimates of expected value of incremental net monetary benefit for blocks 2, . . . ,25.

5. Use the point estimates of the expected value of incremental net monetary benefit for each block and the block sizes, together with Bayesian updating (normal prior and likelihood and known variance) to calculate a path of the prior/posterior mean of expected value of incremental net monetary benefit.

Appendix B Choices of parameter values

The parameter values used for the analysis and presented in Table 1 of the main manuscript are sourced and calculated as follows:

1. Estimate of fixed costs of adopting hydroxychloroquine: these were estimated to be zero.

2. Estimate of the sampling standard deviation, σ_X. We considered two possible values. The first is based on the complete cases and is equal to £7632. The second obtains an estimate from the multiple imputation analysis by using Rubin's rules and is equal to £7615. In view of the facts that: 1. the two values are so similar and 2. there is a large amount of missing data in the study, we used the latter value.

3. Estimate of P, the number of patients affected by the adoption decision. This requires an estimate of the incidence rate of the condition and the time horizon over which the decision will apply. P may then be estimated according to:

$$P = \sum_{i=0}^{T} \frac{I_t}{(1+\delta)^t}, \tag{1}$$

where T is the time horizon and δ is the discount rate [7, 8, 9].

We consulted a range of publications to estimate I_t [10, 11, 12, 13]. To meet the eligibility criteria for the HERO trial patients needed to be aged 18 or over and have OA of the first carpometacarpal (CMC) joint and symptomatic OA affecting other hand joints [14]. Neither [14, 15] nor [6] provide an estimate of the rate at which patients in the United Kingdom report with such symptoms. Using the NHS Hospital Episode Statistics database [16], [12] found 88,178 diagnoses of OA of the first CMC joint between 2000/2001 and 2017/2018 in England, which equates to an incidence rate of approximately 4,900 per year. Absent information about what proportion of these individuals would have met the study's inclusion criteria, we assumed that half of them would have

Variable	Details	Univariate imputation method
Treatment allocation	Binary (Placebo or HCQ)	Complete
Recruitment site	Nominal (13 levels)	Complete
Age	Age in completed years at baseline	Complete
Sex	Binary (Female or Male)	Complete
Body mass index (BMI)	BMI measured in kg/m^2	Complete
Use of analgesia at baseline*	Binary (Yes, No)	Complete
Cost of HCQ	$\mathbb{R}_{\geq 0}$, £0 for participants allocated Placebo	Complete
Cost of other medications*	$\mathbb{R}_{\geq 0}$, £0 if none	Complete
Baseline NRS score	0 - 10, higher scores indicate greater pain	Linear regression (PMM)
Baseline hand strength	Mean across both hands (lbs)	Linear regression (PMM)
Baseline utility	[6]	Linear regression (PMM)
Month 6 utility	[6]	Linear regression (PMM)
Month 12 utility	[6]	Linear regression (PMM)
Cost of GP appointments* (practice visits)	$\mathbb{R}_{\geq 0}$, £0 if none	Linear regression (PMM)
Cost of GP appointments* (home visits)	$\mathbb{R}_{\geq 0}$, £0 if none	Linear regression (PMM)
Cost of nurse appointments* (practice visits)	$\mathbb{R}_{\geq 0}$, £0 if none	Linear regression (PMM)
Cost of other primary care*	$\mathbb{R}_{\geq 0}$, £0 if none	Linear regression (PMM)
Cost of A&E visits*	$\mathbb{R}_{\geq 0}$, £0 if none	Linear regression (PMM)
Cost of outpatient hospital visits*	$\mathbb{R}_{\geq 0}$, £0 if none	Linear regression (PMM)
Cost of physiotherapy in community*	$\mathbb{R}_{\geq 0}$, £0 if none	Linear regression (PMM)
Cost of occupational therapy in community*	$\mathbb{R}_{\geq 0}$, £0 if none	Linear regression (PMM)
Cost of other care in community*	$\mathbb{R}_{\geq 0}$, £0 if none	Linear regression (PMM)

Appendix Table.1: Details of variables included in imputation model used for the HERO trial base case analyses and the analyses reported in this paper (* = medication/appointments relating to hand pain/hand osteoarthritis symptoms only)

done. This gives an estimate of $I_t = 2450$. For the time horizon and discount rate, we followed the approach taken by [17] and set $T = 10$ and $\delta = 0$, so that $P = 24500$.

4. Estimate of c, the marginal cost per pairwise allocation, is calculated using the financial records from the trial. Approximately £90,216 was spent prior to recruiting the first patients. This is classified as the fixed set-up cost of the trial. An estimated 50% of the £409,161 of costs incurred between the start of recruitment and the end of follow-up is taken to be the variable cost of the trial, giving an estimate of the marginal cost per pairwise allocation of £204,580/124 = £1,650. The remaining 50% is taken to be a cost (such as overheads) which would have been incurred during the recruitment phase even if no patients were being recruited. Finally, costs of £336,042 were incurred post follow-up. This gives a total spend of £835,419.

5. Estimate of τ, the delay (measured by the number of pairwise allocations) in observing cost-effectiveness at one year. The trial recruited 248 patients (124 patient pairs) over a period of 611 days (between 24 September 2012 and 27 May 2014). So one pair of patients was randomised approximately every 5 days. This equates to a value of $\tau \approx 74$ pairwise allocations in one year. Comparison of this estimate with the recruitment profile in [17] shows reasonable agreement (the actual number recruited in the first year was approximately 149).

Appendix C Sequential HERO trial cost-effectiveness data

Columns (3), (4) and (5) of Appendix Table 2 provide point estimates of incremental Costs, QALYs and net monetary benefit at one year as evidence accumulated (arranged into blocks of 10 pairwise allocations unless stated otherwise). These estimates are based on the multiply imputed data and are cumulative (e.g. the estimates reported for block 10 are based on the observed/imputed data from the 100 pairs observed up to and including this block). The figures in column (6) are estimates of the expected value of incremental net monetary benefit for each individual block. Column (7) gives the path of the posterior mean of the expected value of incremental net monetary benefit calculated by combining the by block estimates of incremental net monetary benefit given in column (6) (including the prior) using Bayes rule.

(1) Block	(2) Number of pairs	(3) Average incremental QALY (cumulative)	(4) Average incremental cost (£) (cumulative)	(5) Average INMB (£) (cumulative)	(6) INMB (£) (for block)	(7) Posterior mean for E[INMB] (£)
0 (prior)	2	-	-	-	0	0
1+2	20	−0.0659	195.448	−2172.15	−2172.15	−1974.68
3	10	−0.0480	205.335	−1644.59	−423.41	−1489.91
4	10	−0.0386	302.034	−1459.96	427.39	−1033.41
5	10	−0.0181	190.567	−734.59	1420.06	−561.59
6	10	−0.0230	158.461	−849.99	−2029.77	−798.39
7	10	−0.0158	116.218	−590.78	1019.90	−545.85
8	10	−0.0182	151.452	−696.66	894.64	−370.18
9	10	−0.0081	119.886	−364.10	726.66	−250.96
10	10	−0.0130	121.334	−511.87	95.21	−217.02
11	10	−0.0100	70.439	−367.92	−1734.95	−352.55
12	10	−0.0056	6.455	−173.51	1682.90	−185.71
13	4	−0.0028	−38.749	−45.06	4719.12	−30.00

Appendix Table.2: Cost-effectiveness data at one year as evidence accumulated (based on multiply imputed data and assuming a maximum willingness to pay of £30,000 per QALY.)

	Average	Standard deviation	Minimum	Maximum
**Maximum sample size = 1000 pairwise allocations**				
Fixed length trial				
Posterior mean for $\mathbb{E}$[INMB]	–46.69	240.14	–1099.21	800.21
Value-based sequential model				
Posterior mean for $\mathbb{E}$[INMB]	–68.88	512.87	–2425.19	2427.70
Sample size (pairwise allocations)	640.37	282.35	94	1000
**6 months' follow-up**				
Maximum sample size = 124 pairwise allocations				
Fixed length trial				
Posterior mean for $\mathbb{E}$[INMB]	–5.65	334.57	–1264.84	1063.29
Value-based sequential model				
Posterior mean for $\mathbb{E}$[INMB]	–7.78	373.26	–1766.26	1681.58
Sample size (pairwise allocations)	120.01	11.27	57	124
Maximum sample size = 248 pairwise allocations				
Fixed length trial				
Posterior mean for $\mathbb{E}$[INMB]	0.41	238.97	–881.22	825.20
Value-based sequential model				
Posterior mean for $\mathbb{E}$[INMB]	–2.19	338.39	–1766.26	1681.58
Sample size (pairwise allocations)	218.19	47.44	57	248

Appendix Table.3: Comparison of the performance of the designs used for the sensitivity analysis.

	Final decision	
	Hydroxychloroquine not cost-effective	Hydroxychloroquine cost-effective
**Maximum sample size = 1000 pairwise allocations**		
Fixed length trial	0.576	0.424
Value-based sequential model	0.571	0.429
**6 months' follow-up**		
Maximum sample size = 124 pairwise allocations		
Fixed length trial	0.509	0.491
Value-based sequential model	0.508	0.492
Maximum sample size = 248 pairwise allocations		
Fixed length trial	0.500	0.500
Value-based sequential model	0.500	0.500

Appendix Table.4: Sensitivity analysis: proportion of re-sampled paths which suggest that hydroxychloroquine is cost-effective, for the designs summarised in Appendix Table 3.

Appendix D Sensitivity analyses

Appendix D.1 Results of analysis increasing $T_{\max}$

To investigate the operating characteristics of the value-based sequential model in the context of a weak cost-effectiveness signal, but a large maximum possible sample size, we simulated

5000 paths for the following values of $T_{\max}$: 250, 500, 750, 1000, 1500, 2000, 2500, 3000, 4000 and 5000 pairwise allocations. These were generated by sampling values of incremental net monetary benefit from a $\mathcal{N}(\mu_X, \sigma_X)$ distribution, where μ_X = -£45.06 (the final point estimate of the expected value of incremental net monetary benefit based on the imputed data - see Appendix Table 2) and σ_X = £7615 (as per Table 1 of the main manuscript). These sampled values were used to derive the paths of the posterior mean of expected value of incremental net monetary benefit, assuming the same prior distribution as specified in Table 1 of the main manuscript and the same sequence of interim analyses as used for the main analyses (i.e. every 10 pairwise allocations except for the first block of 20 pairs). The change in expected sample size as a function of $T_{\max}$ is plotted in Figure 6 of the main manuscript. Appendix Table 3 reports the operating characteristics for the $T_{\max} = 1000$ case in terms of the final estimates of the expected value of incremental net monetary benefit, and for the value-based sequential model, the number of pairwise allocations made. Appendix Table 4 reports the proportion of simulated paths which conclude in favour of hydroxychloroquine again for $T_{\max} = 1000$.

Appendix D.2 Results using 6 month cost-effectiveness outcomes

Appendix Table 3 reports the average value and standard deviation of the posterior mean at stopping for both the fixed and value-based sequential designs, together with the average sample size (and standard deviation of sample sizes) for the value-based sequential design, assuming a maximum sample size of 124 pairwise allocations and 248 pairwise allocations, based on the cost-effectiveness data at 6 months post-randomisation. Appendix Table 4 reports the proportion of paths which show hydroxychloroquine to be cost-effective, assuming a maximum sample size of 124 pairwise allocations and 248 pairwise allocations, using the cost-effectiveness data observed at 6 months.

Appendix E Assessing the HERO trial's performance using Bayesian value of information metrics

Table 5 presents alternative metrics for evaluating the performance of the value-based sequential model, based on Bayesian value of information criteria.[1] The rows of the table record various different designs of the HERO trial, based on a prior mean for the expected value of incremental net monetary benefit equal to £0 and the parameter values specified in Table 1 of the main paper. The rows list alternative ways of running the trial: row 1 is the original HERO trial design, with a maximum sample size of 124 pairwise allocations (column 2); row 2 is a design whose sample size maximises the Bayesian expected net benefit of sampling and rows 3

[1] We thank an anonymous reviewer for their suggestion to add this material.

(1) Trial design	(2) (Maximum) number of pairwise allocations	(3) Expected net benefit (£)	(4) Percentage increase in expected net benefit
1. HERO trial	124	5.20×10^7	-
2. Value-based one stage model	177	5.20×10^7	0.067
3. Value-based sequential model	124	5.20×10^7	0.066
4. Value-based sequential model	177	5.21×10^7	0.252
5. Value-based sequential model	354	5.22×10^7	0.451

Appendix Table.5: Performance of alternative trial designs for HERO from a Bayesian value-based perspective. (Source: this table is a summary of Table 3.1, page 60, of [17]).

to 5 are three alternative value-based sequential designs whose maximum sample sizes, $T_{\max}$, are displayed in column 2. The first sets $T_{\max}$ to the sample size of the HERO trial, the second to the sample size which maximises the expected net benefit of sampling (row 2), the third doubles this. Column 3 records the total expected net benefit delivered to the healthcare system of each trial design and Column 4 records the percentage increase in expected net benefit of the relevant value-based model over the expected net benefit delivered by the HERO trial (row 1). Full discussion of these metrics, as well as a more detailed table of results, may be found in Chapter 1 and Table 3.1, page 60, of [17].

References

[1] White IR, Royston P, Wood AM. Multiple imputation using chained equations: Issues and guidance for practice. Statistics in Medicine. 2011;30(4):377–399. 1

[2] Royston P. Multiple imputation of missing values. Stata Journal. 2004;4(3):227–241. 1

[3] Rubin DB. Inference and missing data. Biometrika. 1976;63:581–592. 2

[4] Rubin D, Schenker N. Multiple imputation from random samples with ignorable non-response. Journal of the American Statistical Association. 1986;81(394):366–374. 2

[5] Rubin DB. Multiple Imputation for Nonresponse in Surveys. New York: Wiley; 1987. 2

[6] Ronaldson SJ, Keding A, Tharmanathan P, Arundel C, Kingsbury SR, Conaghan PG, et al.. Cost-effectiveness of hydroxychloroquine versus placebo for hand osteoarthritis: economic evaluation of the HERO trial; 2021. F1000research. 2, 3

[7] Claxton K, Posnett J. An economic approach to clinical trial design and research priority-setting. Health Economics. 1996;5:513–524. 2

[8] Philips Z, Claxton K, Palmer S. The half-life of truth: what are appropriate time horizons for research decisions? Medical Decision Making. 2008;28:287–299. 2

[9] Rothery C, Strong M, Koffijberg H, Basu A, Ghabri S, Knies S, et al. Value of Information Analytical

Methods: Report 2 of the ISPOR Value of Information Analysis Emerging Good Practices Task Force. Value in Health. 2020;23(3):277–286. 2

[10] Yu D, Peat G, Bedson J, Jordan K. Annual consultation incidence of osteoarthritis estimated from population-based health care data in England. Rheumatology. 2015;54:2015–2060. 2

[11] Yu D, Jordan K, Bedson J, Englund M, F Blyth AT, et al. Population trends in the incidence and initial management of osteoarthritis: age-period-cohort analysis of the Clinical Practice Research Datalink, 1992–2013. Rheumatology. 2017;56:1902–1917. 2

[12] Morgan OJ, Hillstrom HJ, Ellis SJ, Golightly YM, Russell R, Hannan MT, et al. Osteoarthritis in England: Incidence Trends From National Health Service Hospital Episode Statistics. ACR Open Rheumatology. 2019;1(8):493–498. 2

[13] Swain S, Sarmanova A, Mallen C, Kuo C, Coupland C, Doherty M. Trends in incidence and prevalence of osteoarthritis in the United Kingdom: findings from the Clinical Practice Research Datalink (CPRD). Osteoarthritis and Cartilage. 2020;28:792–801. 2

[14] Kingsbury SR, Tharmanathan P, Adamson J, et al. Hydroxychloroquine effectiveness in reducing symptoms of hand osteoarthritis (HERO): study protocol for a randomized controlled trial. Trials. 2013;14(64). 2

[15] Kingsbury SR, Tharmanathan P, Keding A, et al. Hydroxychloroquine effectiveness in reducing symptoms of hand osteoarthritis: a randomized trial. Annals of Internal Medicine. 2018;168:385–395. 2

[16] Department of Health and Social Care. Hospital Episode Statistics Database; 2020. http://www.webarchives.nationalarchives.gov.uk. 2

[17] Forster M, Flight L, Corbacho B, Keding A, Tharmanathan P, Welch C, et al. Report for the EcoNomics of Adaptive Clinical Trials (ENACT) project : Application of a Bayesian Value-Based Sequential Model of a Clinical Trial to the CACTUS and HERO Case Studies (with Guidance Material for Clinical Trials Units). The University of Sheffield: White Rose Research Online, https://eprints.whiterose.ac.uk/180084/; 2021. 4, 8
